# Familial Hypercholesterolemia in Women: Diagnosis, Treatment, and Cardiovascular Outcomes Across the Lifespan

**DOI:** 10.1007/s11883-026-01431-1

**Published:** 2026-06-08

**Authors:** Irene Karungi, Kirsten B. Holven, Kausik K. Ray, Amany Elshorbagy

**Affiliations:** 1 Department of Primary Care and Public Health, School of Public Health, Imperial College London, London, UK; 2 Department of Nutrition, Institute of Basic Medical Sciences, University of Oslo, Oslo, Norway; 3 Norwegian National Network on Familial Hypercholesterolemia, Oslo University Hospital, Oslo, Norway; 4 Department of Physiology, Faculty of Medicine, University of Alexandria, Alexandria, Egypt

**Keywords:** Women, Familial hypercholesterolemia, Low-density lipoprotein cholesterol, Atherosclerotic cardiovascular disease, Lipid lowering therapy, Pregnancy

## Abstract

**Purpose of Review:**

Familial hypercholesterolemia (FH) is a common monogenic lipid disorder, characterized by lifelong elevated low-density lipoprotein cholesterol (LDL-C) and a markedly increased risk of atherosclerotic cardiovascular disease (ASCVD). Although the genetic prevalence of FH does not differ between sexes, women with FH face unique challenges across their lifespan that contribute to their cardiovascular risk. This review synthesizes current evidence on the trajectory of FH in women, with a focus on disparities in diagnosis, treatment, and cardiovascular outcomes across the female lifespan.

**Recent Findings:**

Current evidence highlights a substantial gender gap in FH care. Compared with men, women are typically diagnosed 3–7 years later, are 26% less likely to receive lipid-lowering therapy (LLT), and are 37% less likely to achieve guideline-recommended LDL-C targets. Childbearing years are a major vulnerable period, as LLT is usually interrupted during pre-conception, pregnancy, and lactation, resulting in a median loss of 2.3 years of statin-treatment per woman. These treatment-gaps contribute to a disproportionately greater cumulative LDL-C burden in younger women with FH than men. Although premenopausal women retain lower absolute ASCVD risk than men with FH, their excess risk relative to the general female population exceeds the corresponding male disadvantage, and both LDL-C and ASCVD risk rise further after menopause.

**Summary:**

Women with FH face distinct diagnostic and therapeutic challenges requiring sex-specific care. Improving equity in FH care in women necessitates early diagnosis, appropriate LLT intensification, dedicated management across the childbearing years, and expanded research into LLT safety in pregnancy to reduce cumulative LDL-C exposure and long-term ASCVD burden.

## Introduction

Familial hypercholesterolemia (FH) is an autosomal dominant disorder arising from genetic variants that impair the clearance of low-density (LDL) lipoproteins [1, 2]. FH is characterized by markedly elevated LDL cholesterol (LDL-C) from birth and is associated with a 10-fold increased risk of coronary artery disease (CAD) in untreated individuals [3]. The more common form, heterozygous FH (HeFH), results from a single pathogenic allele and affects approximately 1 in 300 individuals worldwide [1, 2]. Homozygous FH (HoFH), the more severe form caused by biallelic pathogenic variants, occurs in 1 in 160,000 to 1 in 300,000 births [4]. Up to 90% of FH cases result from LDL receptor (*LDLR*) variants, although apolipoprotein B-100 (*APOB*) or proprotein convertase subtilisin/kexin type 9 (PCSK9), and rarely, recessive LDLR adaptor protein 1 (*LDLRAP1*) variants, also disrupt LDLR function, leading to elevated LDL-C [4, 5].

Despite the availability of effective lipid-lowering therapies (LLT), FH remains underdiagnosed and undertreated globally [1, 2]. Evidence from the largest registry of FH globally, the FH Studies Collaboration (FHSC), demonstrated that most patients worldwide are not identified until their fifth decade of life, with less than 2% of adult cases diagnosed during childhood [1]. This delay is clinically consequential, as approximately one in six adults already has established atherosclerotic cardiovascular disease (ASCVD) at the time of diagnosis [1]. In women, the burden of FH is further influenced by hormonal and reproductive factors that complicate disease recognition and long-term management [6, 7]. Compared to men, women are diagnosed 3–7 years later, treated less intensively, and are 37% less likely to achieve guideline-recommended lipid targets [1, 6]. Moreover, interruption of LLT during preconception, pregnancy, and breastfeeding leads to an estimated loss of 2.3 median years of statin therapy per woman [7]. Consequently, women may accumulate substantial untreated LDL-C exposure during early and middle adulthood [7–9].

ASCVD risk may accelerate further after menopause, a period characterized by rising LDL-C levels and worsening cardiovascular risk profiles [10, 11], compounding the overall ASCVD burden in women with FH [12, 13]. Despite growing recognition of these concerns, important gaps remain in optimizing FH care across the female lifespan [14]. This review summarizes current evidence on the clinical course of FH in women, emphasizing diagnostic disparities, reproductive and menopausal transitions, and opportunities for targeted strategies to improve long-term cardiovascular outcomes.

### LDL-C Burden and Diagnostic Considerations Across the Lifespan of Women with FH

**Fig. 1:**
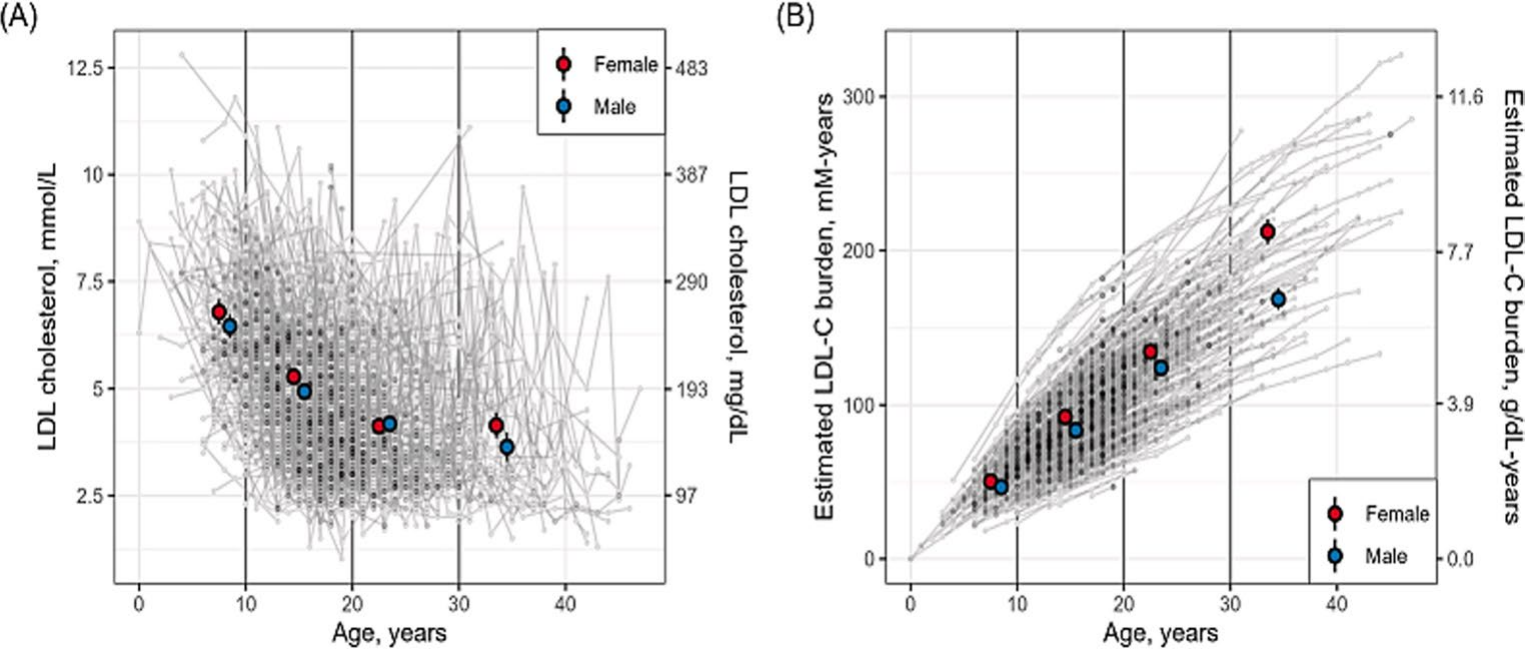
Age and sex-related changes in LDL-C and estimated LDL-C burden. Obtained from Johansen et al. 2023 [9], under the CC BY-NC-ND license. The figure shows measurements of LDL-C (Panel A) and estimated LDL-C burden (Panel B). Individual data points in grey are connected with lines to highlight the subject-specific trends. The figure also shows the sex-specific means and 95% CIs within age strata (< 10 years [*n* = 275], 10–19 years [*n* = 1528], 20–29 years [*n* = 843], and ≥ 30 years [n = 258])

**Fig. 2:**
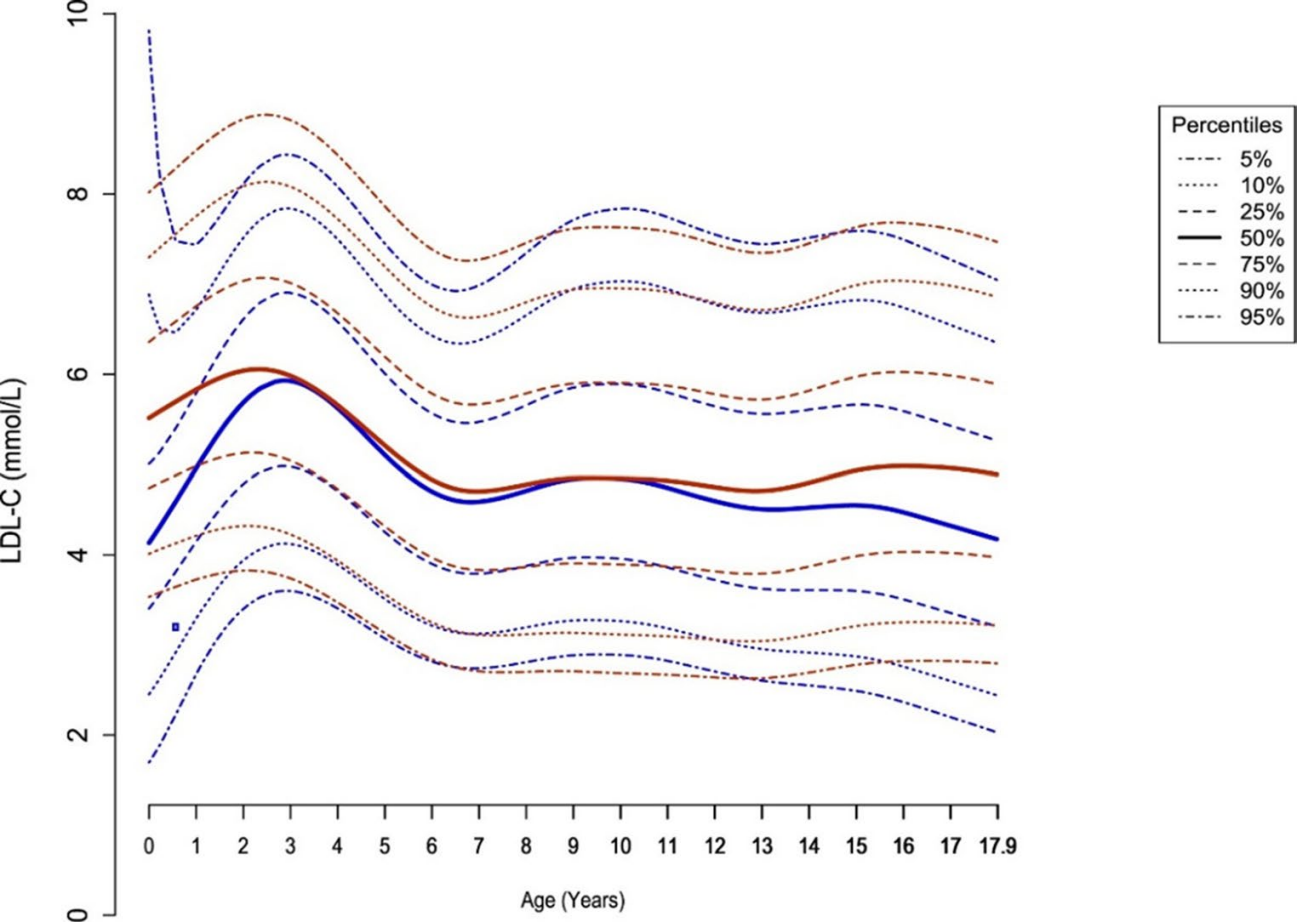
Smoothed percentile curves for LDL-C concentration at entry into the registry among children and adolescents not taking LLT. Blue lines: Untreated male individuals. Red lines: Untreated female individuals. Data are cross-sectional, stratified by age and sex. Adapted from Dharmayat et al. 2024 [17], with permission.

The physiological disadvantage for women with familial hypercholesterolemia (FH) begins long before clinical recognition. While FH is present from birth, untreated girls with FH have higher total cholesterol (TC) and low-density lipoprotein cholesterol (LDL-C) than their male counterparts from birth up to 19 years [8, 15]. This observation may appear counterintuitive, given that premenopausal women in the general population tend to have a more favorable lipid profile than men, partly because endogenous estrogen increases hepatic LDLR expression, thereby enhancing LDL-C clearance [16]. In FH, however, this effect appears insufficient to offset the magnitude of genetically impaired LDL-C metabolism. Indeed, Johansen et al., using repeated LDL-C measurements over a 12-year follow-up of 438 individuals with FH before age 19, demonstrated that the sex difference in the combined pre- and post-treatment LDL-C burden widened over time [9]. By age 19, women with FH were more than twice as likely as men to have exceeded the LDL-C burden threshold of 125 millimolar-years, a level associated with high ASCVD risk [9]. Strikingly, all women had reached this threshold by age 30, whereas it took until the age of 40 for all men to reach the same threshold [9]. Age and sex-related changes in LDL-C and estimated LDL-C burden from this study are shown in Fig. 1. Similarly, cross-sectional data from the FHSC registry [17] for 11,848 children and adolescents with FH suggested that untreated LDL-C concentrations are similar in boys and girls from age 4–10 years but diverge around puberty (from age 11 years onwards), becoming approximately 0.7 mmol/L higher in girls by age 17 years (Fig. 2). In line with our observations in the FHSC [17], a recent study from Slovenia’s Universal FH Screening Program showed no significant differences in LDL-C levels between prepubertal girls and boys with FH aged approximately 5–6 years [18].

After accumulating a substantial LDL-C burden from early in life, women with FH then face additional challenges during their reproductive years. Pregnancy is characterized by marked physiological hyperlipidemia, with a 30–50% increase in LDL-C [19]. In women with FH, who begin pregnancy from a much higher LDL-C baseline, absolute LDL-C concentrations in late gestation may reach levels of 8-9 mmol/L or higher [20]. This period is especially vulnerable because the physiological rise in circulating lipids often coincides with the interruption of therapy, leaving women exposed to prolonged periods of uncontrolled LDL-C concentrations [7, 20]. Furthermore, the physiological stress of pregnancy may contribute to raised blood pressure and dysglycaemia, which may be harbingers of future risk of hypertension and diabetes, and thus may additionally contribute towards atherosclerosis [21, 22].

Beyond the reproductive years, menopause is another critical period in the life course of women with FH [11]. In a Canadian cohort, LDL-C levels increased by 12% overall during menopausal transition, with a greater increase among women with an FH-causing variant (1.23 mmol/L) than in those without (0.39 mmol/L) [11]. Similarly, among adult participants from the FHSC registry, women aged 50 or 55 years or older had LDL-C levels that were, on average, about 0.6 mmol/L higher than those of men (p value < 0.0001) [1]. These findings suggest that menopause may further exacerbate cumulative LDL-C exposure and accelerate ASCVD risk in women with FH [10, 11]. Taken together, these observations highlight how hormonal transitions across the female life course shape lipid biology, and longitudinal data capturing LDL-C trajectories and associated ASCVD outcomes in women with FH remain a key area for future research.

Despite the early and progressive accumulation of LDL-C burden, women with FH are consistently diagnosed later than men [1, 23–26]. This diagnostic delay is about 3–7 years in women [1, 23–25], although one Vietnamese study reported a delay of up to 11 years among female index cases [26]. This pattern is particularly concerning, given that diagnosis of FH already tends to occur late in life for both sexes, frequently only after ASCVD events have occurred [1, 24]. Current diagnostic criteria, such as the Dutch Lipid Clinic Network (DLCN), the Simon Broome Register, and the Make Early Diagnosis to Prevent Early Deaths (MEDPED), do not incorporate sex-or age-specific adjustments, and their performance remains an important area for research [27]. Where genetic testing is available, it provides a definitive diagnosis independent of clinical criteria; however, access remains highly variable globally, with cost and availability representing significant barriers in many healthcare systems [28]. Cascade screening of first-degree relatives is the most cost-effective strategy for identifying new cases [29]. However, the delayed diagnosis in women compared to men inherently delays the screening of their children and siblings. Overall, given the consequences of delayed diagnosis, including greater cumulative LDL-C burden, delayed cascade screening, and hence increased ASCVD risk for both the woman with FH and her undiagnosed relatives, heightened awareness among healthcare professionals is needed to ensure women with FH are identified and treated as early as possible to avoid the cascade of harm resulting from “the law of unintended consequences”.

#### Treatment Strategies Across the Life Span of Women with FH

The goal of FH management is to reduce the future risk of ASCVD, primarily through early diagnosis, effective LDL-C lowering, and management of traditional ASCVD risk factors. For high-risk patients, such as those with FH, current guidelines set by the European Society of Cardiology (ESC) and the European Atherosclerosis Society (EAS) recommend LDL-C targets of < 1.4 mmol/L for adults with ASCVD or FH and one additional risk factor and < 1.8 mmol/L for those without ASCVD, alongside a minimum 50% LDL-C reduction from untreated levels [30]. Although trial data demonstrate that men and women with FH appear to derive similar responses to lipid-lowering therapy (LLT) [6], a previous analysis from our group of 42,167 patients with FH across the 6 WHO regions showed that women were 37% less likely to achieve guideline-recommended LDL-C goals [1]. This likely reflects differences in prescribing patterns for LLT rather than therapeutic responses between men and women.

Statins remain the cornerstone of treatment for both children and adults with FH and provide LDL-C reductions of 30–50% at high doses using more potent regimens [31, 32]. In children, European guidelines recommend starting statin therapy around 8–9 years of age, with ezetimibe added from age 10 if targets are not met [33]. In adults as well as children, ezetimibe is the second-line agent of choice, providing an additional 24% reduction in LDL-C [34]. For patients requiring greater LDL-C lowering, PCSK9 inhibitors reduce LDL-C by 57–67% in HeFH [35, 36], although responses in HoFH may vary depending on residual LDLR activity [37, 38]. Treatment options for HoFH have been expanded by evinacumab, which lowers LDL-C by 47% independent of the LDLR pathway [39, 40]. Bempedoic acid may be a useful option in cases of statin intolerance [41], especially for women, who are more likely than men to report statin-muscle-related symptoms (31% vs. 26%, *p* < 0.01) [42].

Despite advances in LLT availability for FH (Table 1), a meta-analysis of 36 observational studies (129,441 subjects) indeed showed that women with FH are 26% less likely than men to be receiving any LLT, and 30–34% less likely to receive high-intensity statins, ezetimibe, PCSK9 inhibitors, or combination therapy. Additionally, women were 22% less likely than men to attain a 50% LDL-C reduction, and 46% less likely to reach LDL-C values < 1.8 mmol/L [6]. Management for women with FH becomes even more challenging during preconception, pregnancy, and breastfeeding, when most LLTs are contraindicated or not recommended [47]. Oral LLTs such as statins, ezetimibe, and bempedoic acid are generally discontinued approximately one month before actively trying to conceive, while PCSK9 monoclonal antibodies require discontinuation at least three months beforehand, and Inclisiran potentially 9–12 months in advance [45]. Statins have been classified by the United States Food and Drug Administration (FDA) as contraindicated (X) during pregnancy for decades. In 2021, the FDA requested the removal of the longstanding warning, acknowledging that the benefits may outweigh the risks in a small group of very high-risk patients, but still advised that most pregnant patients should stop statins [44, 60]. By contrast, the European Medicines Agency has not made a comparable regulatory change and continues to list statins as contraindicated during pregnancy [60]. As such, the only available therapy options for women with FH are lipoprotein apheresis or bile acid sequestrants such as cholestyramine or colesevelam [47]. Lipoprotein apheresis is highly effective with 60–70% LDL-C reduction; however, it is expensive, requires multiple invasive sessions, and is only available at specialized centers, creating a significant barrier to care for women [47]. Bile acid sequestrants have the advantage of oral intake but have modest LDL-C-lowering efficacy (15–20%) and are often poorly tolerated due to gastrointestinal side effects [47, 61].

**Table 1:** Available and emerging lipid-lowering therapies for patients with FH and their considerations in pregnancy

Drug/Therapy	Approval status in humans	Mechanism of Action	LDL-C reduction (HeFH)	LDL-C reduction (HoFH)	FDA 2018 class for pregnancy†	Consideration for pregnancy among high-risk patients
LDLR Dependent Treatments						
Statins	Approved	Inhibits HMG-CoA reductase, reducing hepatic cholesterol synthesis, thus upregulating LDLR expression	30–50% [32]	14–31% [43]	X designation removed. But contraindication remains. [44]	Generally contraindicated. Only consider use after the 1 st trimester via individualized shared decision-making [45].
Ezetimibe	Approved	Inhibits intestinal cholesterol absorption via NPC1L1 transporter	16–19%* 39–56%^¶^ [34]	20%^¶^ [46]	Was C (Currently NA) [47]	No pregnancy safety data.
Evolocumab	Approved	mAb inhibiting PCSK9, preventing LDLR degradation	60–65% [35]	30% [37]	NA	No pregnancy safety data
Alirocumab	Approved	mAb inhibiting PCSK9, preventing LDLR degradation	57–67% [36]	20–36% [38]	NA	No pregnancy safety data
Inclisiran	Approved	siRNA targeting hepatic PCSK9 mRNA, reducing PCSK9 synthesis, preventing LDLR degradation	41–56% [48]	7–20% [48]	NA	No pregnancy safety data
Bempedoic acid	Approved	Inhibits ATP-citrate lyase upstream of HMG-CoA reductase	24%^¶^ [41]	NA	NA	No pregnancy safety data
Bile acid sequestrants	Approved	Bind bile acids in the gut, interrupting enterohepatic circulation and thus increasing LDLR expression	12–18% [49]	Limited benefit [50]	C [47]	Approved during pregnancy
Lerodalcibep (LIB003)	Phase III	Small recombinant PCSK-binding fusion protein, preventing LDLR degradation	58–65% [35]	4.9% [51]	NA	No pregnancy safety data
Enlicitide (MK-0616)	Phase III	Oral macrocyclic peptide inhibiting PCSK9, preventing LDLR degradation	58% [52]	NA	NA	No pregnancy safety data
AZD8233 (ION449)	Phase II/III	Antisense oligonucleotide targeting hepatic PCSK9 mRNA	39–79% [53]	NA	NA	No pregnancy safety data
CRISPR gene editing	Phase I/II	In vivo base editing of hepatic PCSK9, permanently reducing PCSK9 production	~ 55% [54]	NA	NA	No pregnancy safety data
LDLR Independent Treatments						
Evinacumab	Approved (HoFH)	Monoclonal antibody to ANGPTL3	NA	43–47% [39, 40]	NA	No pregnancy safety data
Lipoprotein apheresis	Approved	Extracorporeal removal of apoB-containing lipoproteins	58–63% (per session) [55]	57–75% (per session) [55]	NA	Considerations of benefits vs. risk should be made
Lomitapide	Approved (HoFH)	Oral inhibitor of microsomal triglyceride transfer protein	NA	35–45% [56]	X [47]	No pregnancy safety data
Mipomersen	Approval withdrawn (2019)	Antisense oligonucleotide targeting apoB-100 mRNA	28% [57]	25% [58]	B (withdrawn)	No longer marketed
Vupanorsen	Discontinued (2022)	Antisense oligonucleotide targeting ANGPTL3	NA	NA	NA	No pregnancy safety data
ARO-ANG3	Phase II/III	siRNA targeting ANGPTL3	38% [59]	NA	NA	No pregnancy safety data
AAV-mediated LDLR gene replacement	Investigational (early phases)	Liver-directed gene therapy delivering a functional copy of the LDLR gene via adeno-associated viral vector	NA	NA	NA	No pregnancy safety data

*LDL-C reduction when therapy is used as monotherapy

^¶^LDL-C reduction when therapy is used as an add-on to other LLT/statin

†FDA pregnancy categories: B: Animal reproduction studies have failed to demonstrate risk to the fetus, and there are no adequate and well-controlled studies in pregnant women. C: animal reproduction studies have shown an adverse effect on the fetus, and there are no adequate and well-controlled studies in humans, but potential benefits may warrant use of the drug in pregnant women despite potential risks. X: studies in animals or humans have demonstrated fetal abnormalities and/or there is positive evidence of human fetal risk based on adverse reaction data from investigational or marketing experience, and the risks involved in use of the drug in pregnant women clearly outweigh potential benefits

*AAV* adeno-associated virus, *ACL* ATP-citrate lyase, *ANGPTL3* angiopoietin-like protein 3, *apoB* apolipoprotein B, *ASO* antisense oligonucleotide, *CVOT* cardiovascular outcomes trial, *FDA* Food and Drug Administration, *HeFH* heterozygous familial hypercholesterolemia, *HMG-CoA* 3-hydroxy-3-methylglutaryl-coenzyme A, *HoFH* homozygous familial hypercholesterolemia, *LDL* low-density lipoprotein, *LDL-C* low-density lipoprotein cholesterol, *LDLR* low-density lipoprotein receptor, *LPL* lipoprotein lipase, *mAb* monoclonal antibody, *MTP* microsomal triglyceride transfer protein, *NA* no data available (not reported), *NPC1L1* Niemann-Pick C1-Like 1, *PCSK9* proprotein convertase subtilisin/kexin type 9, *RCT* randomized controlled trial, *siRNA* small interfering RNA, *VLDL* very low-density lipoprotein

The consequences of the time lost in therapy are substantial. A study of 102 women from Norway and the Netherlands found that although the median pregnancy-related off statin period was 2.3 years, some women experienced off-treatment periods of up to 14 years, corresponding to a loss of 20% of lifetime statin treatment years [7]. When untreated years in childhood and prior to diagnosis are also factored in, women with FH spent a median of 66.3% (41.9–100%) of their lifetime without LLT [62]. Notably, 22% of women with FH who breastfed reported stopping earlier than desired in order to restart statin therapy, and 86% of them reported needing more information about pregnancy and breastfeeding in the context of FH [7]. A recent review addressing these gaps presents an updated meta-analysis confirming that statin exposure in pregnancy is not associated with increased congenital malformations (OR 1.03, 95% CI 0.89–1.18). As such, the authors recommend individualized consideration of statin continuation after the first trimester in high-risk women, such as those with established ASCVD or HoFH, following shared decision making [45]. The same review highlights that women with FH face an increased risk of pre-eclampsia during pregnancy, which itself is associated with increased long-term CVD risk, further underscoring the need for enhanced monitoring during pregnancy. Regarding breastfeeding, reintroducing LLT at 6–12 months postpartum represents a pragmatic approach to minimizing cumulative LDL-C exposure without unduly compromising breastfeeding benefits [45]. Even so, high-quality prospective data on LLT therapy during pregnancy and breastfeeding remain limited and represent a pressing research need.

Although elevated LDL-C is the key driver of ASCVD in FH, this risk is further amplified by traditional risk factors, including obesity, type 2 diabetes, hypertension, and smoking. In a recent US multicenter study of 782 FH patients, hypertension was a stronger predictor of premature ASCVD than smoking or diabetes in both sexes, with slightly higher odds in women (4.25) than men (3.83) [63]. Our FHSC analysis of 29,265 adults with HeFH demonstrated that globally, overweight was more common in men (42%) than in women (30%), whereas obesity was slightly more prevalent in women (17.2% vs. 15.4%). Importantly, the association between obesity and CAD was similar in men and women, underscoring the need for equally aggressive weight management in both sexes [64]. Obesity was also associated with fivefold higher odds of type 2 diabetes in the pooled cohort, but women remained 19% less likely than men to have diabetes, after adjusting for age, BMI, and LLT [65], as reported in a smaller cohort [66]. Overall, these findings highlight that sex may need to be considered in the personalized management of traditional cardiometabolic risk factors in FH to reduce ASCVD risk.

## Burden of ASCVD in Women with FH

Despite the reported apparent better LDL-C control and fewer treatment interruptions in men, our FHSC data from over 42,000 adults showed that, compared to men (median age 45 years), women (median age 48 years) had approximately half the prevalence of CAD (12% vs. 21%) and premature CAD (7% vs. 15%) [1]. These differences are consistent with findings from other FH cohorts, all of which report lower cardiovascular morbidity in women with FH than in men [23–25]. Among the elderly, a Brazilian FH cohort also showed that male sex was independently associated with ASCVD (OR 2.67 (95% CI 1.50–4.73)) [67], as observed in non-elderly patients [1, 23–25]. Similarly, the Spanish FH cohort study also found that female sex was independently associated with cardiovascular resilience among those who survived to older age [68]. With regards to the pathophysiology underlying overt ASCVD, in a recent multinational study of 1,011 individuals with FH, most of whom were genetically diagnosed and receiving statins, women were less likely than men to present a subclinical coronary atherosclerosis burden across all ages [69]. A coronary calcium score of zero, a robust negative marker for ASCVD event risk, was more frequent in women than men (48% vs. 35%), as was the absence of plaques on computed tomography angiography (39% vs. 26%) [69].

On the other hand, women with FH appear to experience greater excess cardiovascular burden relative to the general population than men. In 3,553 FH individuals in the UK Simon Broome register the standardized morbidity ratio was 50% higher in women than men among those aged 30–50 years and 33% higher in those aged > 50 years [24]. Additional data from the same registry showed persistent sex differences in coronary mortality ratio over time. Among men with definite FH and established ASCVD, the excess coronary mortality ratio declined over the years from 4.83 (95% CI 2.32–8.89) in pre-1992 to 2.51 (95% CI 1.01–5.17) post-2008, whereas in women, the corresponding values were 7.23 (2.65–15.73) and 6.34 (2.06–14.81) [13]. A similar pattern was observed in Norway, where standardized mortality rates compared with the general population were 2.00 (95% CI 1.32–3.04) in men and 3.03 (95% CI 1.76–5.21) in women [70]. Taken together, these data indicate that FH confers a greater excess cardiovascular risk in women than in men relative to the general population, even though men with FH retain higher absolute event rates. This disproportionate impact of FH on women may be driven by their higher cumulative LDL-C burden related to later diagnosis, less intensive lipid-lowering therapy, and treatment interruptions during pregnancy and breastfeeding, all of which minimize the cardiovascular protection that unaffected women would otherwise experience.

## Future Direction

Given the evidence that women with FH have higher untreated LDL-C than men and experience unique treatment challenges in their reproductive years, as well as further LDL-C elevation around menopause, future research should prioritize the development of sex and age-specific approaches to FH diagnosis and management. In particular, there is a need for prospective studies evaluating how puberty, pregnancy, lactation, and menopause influence not only LDL-C trajectories but also ASCVD outcomes in women. Lastly, greater emphasis should be placed on improving access to genetic testing and generating high-quality safety data for LLT during pregnancy and breastfeeding.

## Conclusion

Women with FH experience a distinct lifespan pattern of LDL-C exposure, shaped by hormonal factors, treatment interruptions, and delayed diagnosis. Although effective LLTs are available, disparities in treatment intensity and the constraints imposed by reproductive years may contribute to disproportionately high excess ASCVD risk in women with FH relative to the general population. A more sex-specific and lifespan-informed approach to FH research and care is needed to improve outcomes in women with FH.

## Key References

- Global perspective of familial hypercholesterolaemia: a cross-sectional study from the EAS Familial Hypercholesterolaemia Studies Collaboration (FHSC). Lancet. 2021;398(10312):1713-25.
  - **○ **This landmark cross-sectional study from the largest global FH registry provided comprehensive sex differences on FH diagnosis, treatment, and outcomes.
- Iatan I, Akioyamen LE, Ruel I, Guerin A, Hales L, Coutinho T, et al. Sex differences in treatment of familial hypercholesterolaemia: a meta-analysis. Eur Heart J. 2024;45(35):3231-50.
  - **○ **This is the largest meta-analysis to date, quantifying sex differences using data from 16 clinical trials and 36 observational studies.
- Holven KB, Raal FJ, Watts GF, Christensen JJ, Roeters van Lennep J. Challenges in the care of women with familial hypercholesterolaemia during the reproductive period; current evidence and practical guidance. Atherosclerosis. 2026;415.
  - **○ **This review provides the most current evidence-based guidance on managing FH during reproductive years, including updated meta-analytic data from 8 studies on statin safety in pregnancy and practical recommendations to minimize treatment gaps in women with FH.
- Elshorbagy A, Lyons ARM, Vallejo-Vaz AJ, Stevens CAT, Dharmayat KI, Brandts J, et al. Association of BMI, lipid-lowering medication, and age with prevalence of type 2 diabetes in adults with heterozygous familial hypercholesterolaemia: a worldwide cross-sectional study. The Lancet Diabetes & Endocrinology. 2024;12(11):811-23.
- Elshorbagy A, Vallejo-Vaz AJ, Barkas F, Lyons ARM, Stevens CAT, Dharmayat KI, et al. Overweight, obesity, and cardiovascular disease in heterozygous familial hypercholesterolaemia: the EAS FH Studies Collaboration registry. European Heart Journal. 2025;46(12):1127-40.
  - **○ **Drawing on the FHSC registry, these cross-sectional studies reveal sex differences in traditional risk factors such as obesity and DM in patients with FH globally, providing important context for sex-specific risk stratification.
- Klevmoen M, Bogsrud MP, Retterstøl K, Svilaas T, Vesterbekkmo EK, Hovland A, et al. Loss of statin treatment years during pregnancy and breastfeeding periods in women with familial hypercholesterolemia. Atherosclerosis. 2021;335:8-15.
  - **○ **This study provides unique real-world data on cumulative loss of statin treatment years due to pregnancy and breastfeeding periods in women with FH.
- Johansen AK, Bogsrud MP, Christensen JJ, Rundblad A, Narverud I, Ulven S, et al. Young women with familial hypercholesterolemia have higher LDL-cholesterol burden than men: Novel data using repeated measurements during 12-years follow-up. Atheroscler Plus. 2023;51:28-34.
  - **○ **Using 12 years of repeated LDL-C measurements, this study provides longitudinal evidence that young women with FH accumulate a greater LDL-C burden than men.

## Data Availability

No datasets were generated or analysed during the current study.
